# Demonstration of sub-luminal propagation of single-cycle terahertz pulses for particle acceleration

**DOI:** 10.1038/s41467-017-00490-y

**Published:** 2017-09-04

**Authors:** D. A. Walsh, D. S. Lake, E. W. Snedden, M. J. Cliffe, D. M. Graham, S. P. Jamison

**Affiliations:** 1 Accelerator Science and Technology Centre, Science and Technology Facilities Council, Daresbury Laboratory, Keckwick Lane, Daresbury, Warrington, WA4 4AD UK; 2 The Cockcroft Institute, Sci-Tech Daresbury, Keckwick Lane, Daresbury, Warrington, WA4 4AD UK; 3 School of Physics and Astronomy & Photon Science Institute, The University of Manchester, Manchester, M13 9PL UK

## Abstract

The sub-luminal phase velocity of electromagnetic waves in free space is generally unobtainable, being closely linked to forbidden faster than light group velocities. The requirement of sub-luminal phase-velocity in laser-driven particle acceleration schemes imposes a limit on the total acceleration achievable in free space, and necessitates the use of dispersive structures or waveguides for extending the field-particle interaction. We demonstrate a travelling source approach that overcomes the sub-luminal propagation limits. The approach exploits ultrafast optical sources with slow group velocity propagation, and a group-to-phase front conversion through nonlinear optical interaction. The concept is demonstrated with two terahertz generation processes, nonlinear optical rectification and current-surge rectification. We report measurements of longitudinally polarised single-cycle electric fields with phase and group velocity between 0.77*c* and 1.75*c*. The ability to scale to multi-megavolt-per-metre field strengths is demonstrated. Our approach paves the way towards the realisation of cheap and compact particle accelerators with femtosecond scale control of particles.

## Abstract (web summary)

Controlled generation of terahertz radiation with subluminal phase velocities is a key issue in laser-driven particle acceleration. Here, the authors demonstrate a travelling-source approach utilizing the group-to-phase front conversion to overcome the sub-luminal propagation limit.

## Introduction

Femtosecond duration relativistic electron beams are in demand for ultrafast electron diffraction, as sources of femtosecond X-rays in free-electron laser facilities such as the Linac Coherent Light Source (LCLS)^1^ and SPring-8 Angstrom Compact Free Electron Laser (SACLA)^2^, and for future lepton colliders such as CERN’s proposed 35 km long Compact Linear Collider, CLIC^3^. For over 50 years gigahertz frequency electromagnetic fields have been the drivers in increasingly sophisticated generations of particle accelerators, but face significant challenges in meeting the demands for control and manipulation of particle beams on the femtosecond scale. Physical limits in the achievable acceleration field strengths at gigahertz frequencies also underlies the km-scale and multi-megawatt power consumption of high-energy accelerators.

To overcome the limitations of few-gigahertz frequency acceleration structures many concepts for millimetre-wave and terahertz frequency acceleration have been investigated. Among those that have demonstrated significant potential are beam-driven dielectric lined structures^4, 5^, W-band resonant structures operating at 91 GHz^6^, and high frequency photonic band gap structures^7^. The surface breakdown field strengths at these higher frequencies significantly exceed those of few-GHz radio frequency structures^8^, and have even been observed to exceed 10 GeV m^−1^ in beam-driven dielectric structures^9^. Laser-based approaches have also demonstrated strong potential for high gradient and compact particle acceleration, with laser-plasma wakfield acceleration (LPWFA) producing quasi-monochromatic electron beams through self-injection of electrons from the background plasma^10–13^. Through systematic investigation and improvements over the last decade, LPWFA now has a demonstrated capability of multi-GeV electron acceleration, acceleration gradients exceeding 10 GeV m^−1^ ^14, 15^, and generation of femtosecond duration electron bunches^16–19^. However, despite the very high gradients and acceleration achieved, exploitation of LPWFA is hampered by shot-to-shot stability in the electron beam parameters and by a relatively large energy spread of the accelerated beams. Laser-driven photonic and dielectric structures also have the potential for GeV m^−1^ acceleration gradients^20, 21^, although the small sub-micron structure apertures and few-fs electromagnetic periods present significant challenges in injection, and in controlling wakefields and beam space-charge effects.

Recently there has been significant interest in the use of laser generated terahertz pulses for particle acceleration as a potential new approach with promise to overcome both the limitations of radio frequency (RF) acceleration, and the significant challenges remaining in the other high-frequency concepts. With their picosecond period fields, terahertz sources offer the ability to capture and finely control few-fs particle beams^22–24^, while the few-ps envelope of energy content offers orders of magnitude improvement in matching energy localisation to the charged particles being accelerated. A terahertz acceleration scheme has recently demonstrated the ability to accelerate non-relativistic particles, using a waveguide structure to accelerate electrons from an initial energy of 60–67 keV over a distance of 3 mm^25^. Ultrafast terahertz pulse sources have also recently been shown to produce GV m^−1^ electric field strengths^26–28^, exceeding by an order of magnitude that of normal-conducting RF accelerators^29^, and two orders of magnitude that of superconducting accelerators such as the European XFEL^30^. To obtain continuous and length-scalable acceleration of relativistic particles however, requires phase-matching of the electromagnetic carrier wave with the *β* = v/c < 1 velocity of the particles in order that these high electric field strengths can be exploited.

The highest field strength terahertz sources are obtained in near-single-cycle electromagnetic pulses, containing an exceptionally broadband coherent spectrum. The single-cycle nature of the pulse is both central to its application for particle acceleration, and to-date the principal obstacle to scalable implementation. The single-cycle near transform limited time profile gives rise to the highest field strengths, and the 100 fs to 1 ps carrier period is ideally matched to capture and control particle beams with sub 10 fs duration; additionally the broadband coherence allows for customised electric field temporal profiles, such as linear ramps. With an electromagnetic energy temporally concentrated within 1 ps and well matched to the duration of particle bunches, orders of magnitude efficiency gains over conventional RF acceleration (with the μs and ms fills times of normal and superconducting cavities, respectively) may be achieved.

To obtain acceleration over extended interaction lengths requires generation of electromagnetic modes with longitudinal electric field polarisation, and real or effective phase-matching of the electromagnetic carrier wave with the *β* < 1 velocity of the relativistic particles. Longitudinal polarised terahertz radiation can be obtained through coherent spatial addition of phase or polarity offset beams^31^, through selective coupling into (or onto) geometrically defined structures^32, 33^, using air-plasma generation^34^, using a segmented nonlinear generation crystal^35^, or using radially biased photoconductive antennas^36–40^. The phase-matching problem is however more fundamental; free space beams such as TEM_10_ Gaussian modes necessarily have phase velocities greater than the vacuum speed of light, *c*, with the Guoy *π*-phase shift ultimately limiting available interaction lengths to less than a Rayleigh length^41^. Waveguiding is capable of obtaining *β* < 1 phase-matching at a single frequency (or subset of frequencies) but such structures are necessarily subject to dispersion relations that preclude maintaining the single-cycle field profile over an extended region. For few-cycle (≲10 ps) terahertz pulses, phase-matching and high field strengths can be maintained over longer distances, but they are subject to a further inherent limitation of separation of electromagnetic energy and particle location through group-phase velocity mismatch^42^.

Here, we demonstrate the generation of terahertz frequency electromagnetic fields that are single-cycle, polarised in the direction of propagation, that propagate in vacuum without dispersion, and have phase velocities that can be tuned to less than the velocity of light. The concept of velocity and polarisation control is demonstrated with differing physical mechanisms of terahertz pulse generation, photoconductive antenna and nonlinear optical rectification, and in nonlinear media known to be capable of fields strengths exceeding the 10 MV m^−1^ of start-of-the-art superconducting accelerators. The concept is scalable without limit in interaction length, not being subject to limitations of dispersion, and we show that it is possible to achieve sustained energy gain over an extended distance. It is shown that the transverse forces accompanying the acceleration fields provide guiding focusing fields analogous to those in RF cavities. Our approach will enable the length scalable high-gradient relativistic particle acceleration required for application to femtosecond electron diffraction and for driving compact X-ray free-electron lasers.

## Results

### Sub-luminal travelling wave

**Fig. 1:**
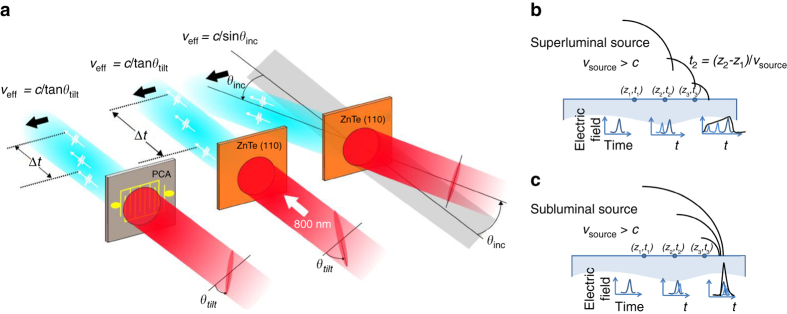
Effective travelling wave concept, for both a nonlinear material and a photoconductive antenna. **a** For a tilted pulse front the effective velocity is unbounded, while for an obliquely incident plane wave the effective source velocity is constrained to be greater than *c*. Tilt and incident angles, *θ*_tilt_ and *θ*_inc_, respectively, are those external to the source material. PCA: photoconductive antenna. **b**, **c** Schematic of Huygens waves (*black*) generating the propagating field in the vacuum region above a dielectric medium. The *shaded regions* represent the dielectric medium. **b** For a super-luminal source velocity the Huygens waves interfere to give a plane wave, while **c** for a single-cycle sub-luminal source interference and cancellation is absent

Laser-driven terahertz sources based on photoconductive antennas (PCA) or optical rectification provide a conversion of the optical pulse (group) arrival time to a terahertz carrier phase. For the PCA the optical energy deposition is responsible for carrier generation, setting the time (phase) of the radiating surface current surge^43^. For optical rectification, the coherent convolution of difference frequency mixing combinations can be shown to produce a terahertz carrier field following the time-derivative of the optical envelope^44^. To achieve a sub-luminal carrier wave, we exploit this group-to-phase conversion together with tilted optical pulse-fronts giving a controllable arrival-time delay exciting a planar terahertz source. Tilted pulse-fronts are generated through imaging of the pulse propagated from an optical diffraction grating. The optical wavefronts of the pulse remain orthogonal to the propagation direction, while there is a transverse time delay in the local energy content of the pulse. As shown in Fig. 1 when impinging on a source plane (a nonlinear *χ* ^(2)^ material or PCA) the delayed optical energy arrival produces an effective travelling wave source for the terahertz radiation. In the case of optical rectification, the preservation of wavefront direction orthogonal to propagation allows the one-dimensional pencil-beam *χ* ^(2)^ phase-matching conditions to be maintained independent of the pulse-front-tilt angle. The use of tilted pulse-fronts is central to obtaining an effective sub-luminal terahertz phase velocity. With pulse-front-tilt spanning the range $0\leqslant {\phi _{{\rm{tilt}}}} < \pi {\rm{/}}2$ the effective surface optical group velocity (and hence terahertz phase velocity) can span $0 < v_{{\rm{group}}}^{{\rm{opt,eff}}}\le \infty$. As the optical carrier wavefront remains travelling with normal angle of incidence with effective surface velocity $v_\phi ^{{\rm{opt,eff}}} = \infty$ it cannot however be exploited directly for acceleration. Although a simpler non-normal incident planar pulse front can also provide effective velocity control, such an arrangement is limited to velocities greater than *c*; due to Snell’s law refraction of the pulse front on entry to the velocity reducing medium, this limitation of planar pulse-fronts holds even when the source is embedded in a material of refractive index *n* > 1.

### Propagating evanescent waves

The excitation of the terahertz sources through tilted optical pulse fronts allows coupling of the optical energy into the source material at sub-luminal velocities. The outcoupling of the terahertz pulse from inside the source material is likewise subject to conditions of boundary continuity and refraction, and for conventional many-cycle electromagnetic waves the transition to sub-luminal source propagation is equivalent to meeting conditions for the critical angle of total internal reflection and post-boundary evanescent wave propagation^45^. For the single-cycle pulses generated by optical rectification the classification of the post-boundary fields as non-propagating and exponentially decaying in amplitude is no longer appropriate. Conceptually describing the travelling wave source as a superposition of time delayed Huygens spherical waves the sub-luminal and super-luminal velocities, and single-cycle vs. multi-cycle pulses, give rise to qualitatively different solutions for the propagating waves. For the super-luminal source (Fig. 1b) a superposition of waves gives rise to a solution with a far-field distribution approximating an obliquely propagating plane-wave satisfying Snell’s law of refraction. For the sub-luminal case the Huygens waves of a single-cycle pulse cannot coincide, with the exception being *v*_*ϕ*_ = *c* for which they add coherently along the surface. For single-cycle pulses the process of constructive or destructive interference for Δ*ϕ* > 2*π* does not arise and the exponential decay of the field with distance from the surface associated with evanescent waves is supplanted with a slower 1/*r* decay of the Huygens fields and a temporal stretching arising from the superposition of wavelets retarded in time by the sub-luminal source velocity.

**Fig. 2:**
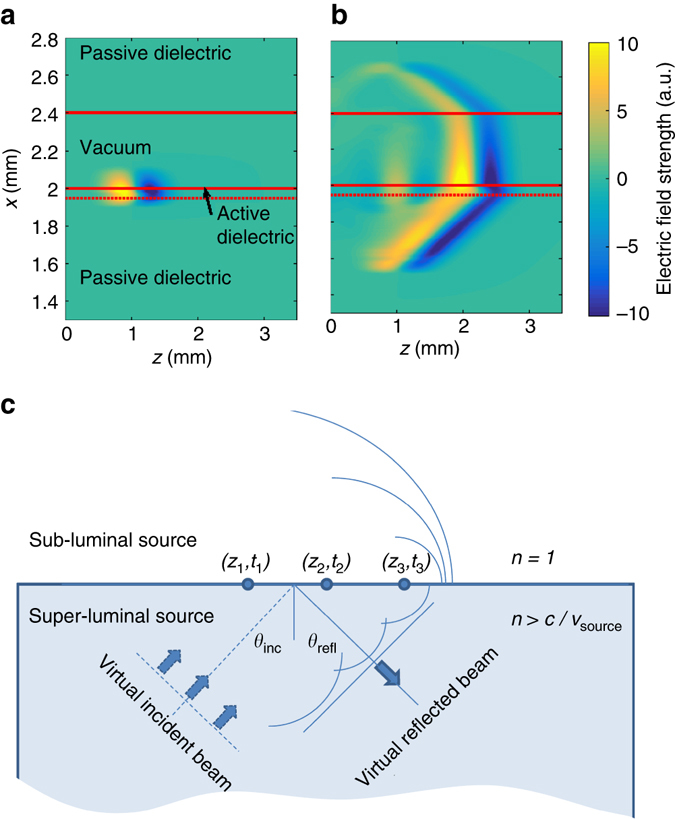
The field evolution for a nonlinear source within a dielectric. **a**, **b** Finite difference time domain calculations of the electric field for a travelling source within a dielectric layer on a dielectric substrate. The terahertz source is only present within a 50 μm active layer. The source velocity is greater than the speed of light within the dielectric, but less than the speed of light in vacuum. Time snap-shots are shown for **a** 0.5 ps and **b** 4.5 ps after the start of the nonlinear interaction. **c** The travelling source can be viewed as a surface excitation driven by a virtual incident plane wave. For a sub-luminal (in vacuum) effective surface velocity the angle of incidence for the virtual wave will exceed the critical angle for total internal reflection. For thicker source regions the virtual source has a real component propagating within the dielectric and spatio-temporal shaping will be required for maximum terahertz generation efficiency

To provide a detailed and quantitative picture of the single-cycle propagation from a sub-luminal source, finite difference time domain (FDTD) simulations have been undertaken (Methods section). An example of finite difference time domain calculation of emission from (and into) the surface under sub-luminal conditions is presented in Fig. 2a. In this example the terahertz radiation is generated only within the top 50 μm layer of the dielectric; such a situation arises, for example, with a (110) oriented and *χ* ^(2)^ active ZnTe layer on a thicker inactive (100) oriented ZnTe substrate. Thinner sub-micron source layers are expected for photoconductive antenna. For thicker source regions spatio-temporal shaping of the source may also be required for high-efficiency generation, as discussed below for LiNbO_3_ sources. After an initial stage of propagation where the field is established in the region above the source plane, a stable pulse is obtained, travelling with a wavefront normal to the surface and with a velocity set by the effective source velocity. The normal-to-surface extention of the field structure, which evolves over a finite time, is responsible for an apparent instananeous propagagtion of the field across a gap in frustrated total internal reflection^45, 46^. The physical connection to total internal reflection is described schematically in Fig. 2b, and is apparent in the numerically calculated field profiles of Fig. 2a. A surface sub-luminal travelling source is analogous to that arising from a virtual planar wavefront arriving at an angle of incidence exceeding the critical angle. This virtual incident beam establishes a real beam propagating as if reflected, together with the sub-luminal fields propagating across the boundary into the vacuum region. The use of total internal refection and evanescent fields has been previously proposed for particle acceleration^47, 48^. While the resulting field structures and evolution is analogous to that expected in total internal reflection, our scheme does not rely on a real (terahertz) incident beam and avoids the associated obstacles of in-coupling of a pulse at a sub-critical angle.

### Observation of single-cycle sub-luminal propagation

**Fig. 3:**
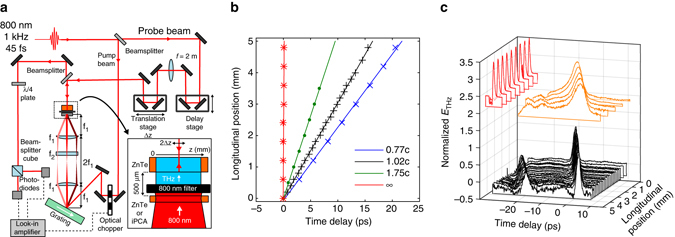
Experimental measurements of the terahertz emission from a travelling source. **a** Experimental arrangement for the spatial and temporal characterisation of the terahertz pulse emitted from a ZnTe or interdigitated photoconductive antenna (iPCA) travelling wave source. **b** World lines of the terahertz pulse measured for a ZnTe source with differing pulse-front tilts. The data points are the time of the peak electric field. The lines are linear fits to the data, and correspond to phase and group velocities from 0.77*c* to 1.75*c*. **c** Examples of the single-cycle terahertz pulse temporal profiles measured at positions in a plane parallel to the emitting surface. The longitudinal direction is defined as the direction of propagation of the travelling wave source. From *top* to *bottom*, the pulses were generated by a planar wavefront optical pulse exciting a ZnTe emitter (*v* ^eff^ = ∞), and *θ*_tilt_ ~ 45° pulse front exciting an PCA and ZnTe emitters

Sub-luminal dispersionless terahertz pulses have been generated using the concepts of Fig. 1, in both a large-area interdigitated photoconductive antenna and through optical rectification in a (110)-cut ZnTe single crystal (see Methods section). The experimental configuration is shown schematically in Fig. 3a. In both cases the optical pulse (group) front tilts were produced by a diffraction grating, with the diffracted pulse imaged onto the generation PCA or ZnTe crystal. The optical pump beam was incident normal to the generation plane. Temporal and spatial characterisation of the field was undertaken with electro-optic sampling with a 50 fs optical probe retro-reflected from the internal boundary of a separate ZnTe electro-optic detection crystal. The terahertz electric field emitted from the source and propagated through air into the detection material effectively creates a birefringence in the detection crystal that temporally and spatially replicates the incident terahertz pulse, and this birefringence is observed through the polarisation change of the optical probe (see Methods section). Figure 3c shows pulse propagation measurements for a normal-incidence planar pulse front optical pump on a ZnTe emitter, with $v_{{\rm{eff}}}^{{\rm{THz}}} = \infty$, and measurements of $v_{{\rm{eff}}}^{{\rm{THz}}} \approx c$ pulse propagation in ZnTe and PCA sources. The tuneability from sub- to super-luminal propagation has been observed with temporal-spatial mapping of the pulse evolution undertaken for a range of pulse-front tilts, corresponding to effective velocities from 1.75*c* down to 0.77*c*. Measured world lines in ZnTe are shown in Fig. 3b. We observe no significant pulse broadening or reshaping as a function of wave propagation along the surface; variations in intensity are attributable principally to the transverse intensity profile of the optical pulse providing the terahertz excitation.

### Application to particle acceleration and manipulation

**Fig. 4:**
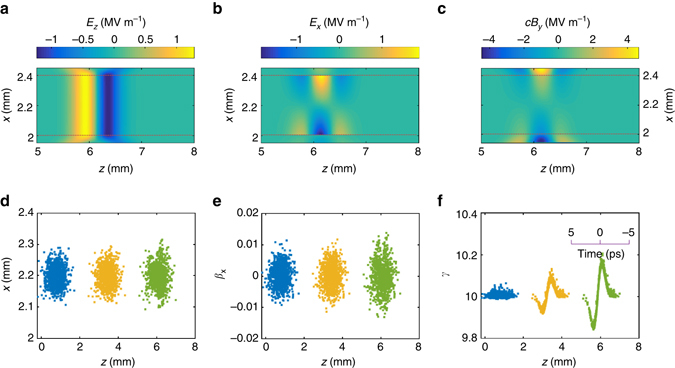
Particle acceleration in a paired travelling source structure. **a**–**c** longitudinal electric field (*E*_*z*_) and transverse electric and magnetic field components (*E*_*x*_, *H*_*y*_) at *t* = 18 ps for a 500 fs, bipolar terahertz source with peak longitudinal field strength of 10 MV m^−1^ generated in each crystal. Nonlinear dielectric media are present at *x* < 2.0 mm and *x* > 2.4 mm, with a vacuum region in between. The vacuum/dielectric interfaces are marked with *dotted lines*. The nonlinear interaction in each crystal generates identical longitudinal field *E*_*z*_ and opposite polarity transverse fields *E*_*x*_, *B*_*y*_. **d**–**f** Transverse position, angle and energy respectively for 1000 electrons after acceleration in a 15 mm structure as a function of particle position in the bunch; particles enter the structure at 5.2 MeV/c, with 500 fs rms duration with 100 μm rms diameter. The *blue*, *yellow* and *green* particle distributions correspond to those at times 0, 8.8 and 17.7 ps, respectively. For a 5 MeV beam energy with particle velocity *β*/*c* = 0.995, there is negligible velocity spread within the bunch, allowing the behaviour of shorter duration bunches to be inferred directly from the corresponding temporal slice in the particle distributions shown

Both PCAs and appropriately oriented *χ* ^(2)^ optical rectification sources will produce terahertz electric fields polarised in the plane of the source. With the field polarisation aligned in the direction of propagation of the travelling wave (the longitudinal direction) co-propagating charged-particle acceleration becomes possible. The longitudinal accelerating electric fields are accompanied by significant transverse electric and magnetic fields which arise through the spatial and temporal gradients of the terahertz source. For a relativistic particle with *β* ≲ *c* the magnetic deflecting force becomes comparable to the accelerating force. Additionally the transverse electric field necessary to satisfy the condition of vanishing electric field divergence in the vacuum gap becomes comparable to the accelerating field temporally ahead and behind the region of acceleration. As in laser-dielectric acceleration schemes, and in conventional RF cavity acceleration, these deflection forces can be eliminated or reduced through imposing additional symmetry around the particle beam-axis. Here, we consider a two-dimension arrangement with sources located in a pair of *z*−*y* planes. This two-dimensional (2D) symmetric arrangement and the resultant field structure is similar to the total internal reflection scheme proposed by Frandsen et al.^47^ and Pálfalvi et al.^48^. Figure 4 shows the electric and magnetic fields, calculated with FDTD modelling, in the vacuum gap between a pair of opposing sources embedded in dielectric media separated by 400 μm (see Methods section). The longitudinal electric field sources *E*_*z*_(*x* = ±250 μm, *z*−*β*_s_ *ct*, *t*) are localised in *z*−*y* planes with *x* = ±250 μm, and propagate with source velocity *cβ*_s_. The source velocity in these calculations was *β*_s_ = 0.995, corresponding to velocity matching for a 5 MeV electron beam. Due to cancellation of the transverse electric and magnetic fields in the symmetry plane of the source-pair, the deflection forces are minimal in a region of ≈100 μm around the central beam-axis, providing an acceleration potential bucket sufficient for injection of particle beams from conventional electron guns. The field structure produced from the opposing pair travelling sources is similar to that found within conventional RF accelerating structures, with the exception that the accelerating fields and electromagnetic energy are co-propagating in synchrony with the charged particles rather than stored over the longer times and spatial extent in an accelerating cavity structure.

The results of modelling particle acceleration of a 5 MeV electron beam injected into the travelling source paired structure are shown in Fig. 4d–f. The particle dynamics has been determined through numerical solution of the relativistic Lorentz force equation, and include the electric and magnetic deflection forces (see Methods section). For the 2D arrangement of the source, the acceleration and deflection occur in the *z* and *x* axes, with *E*_*y*_, *H*_*x*_, *H*_*z*_ = 0 by symmetry. The injected beam has a *σ*_*t*_ = 1 ps root mean square (r.m.s.) pulse duration, and a transverse phase-space emittance of 0.3 mm mrad, consistent with that obtainable from state-of-the-art RF photo-injectors^1, 49^. We show the evolution of the particle distribution as the particles interact over ~7 mm with a peak accelerating gradient of 10 MV m^−1^ and a 500 fs bipolar terahertz field representative of our LiNbO_3_ source to be described in the following section. Net energy gain of ~100 keV is obtained when injecting close to the peak phase of the accelerating field. In contrast to acceleration schemes using optical wavelengths, the approximately 100 μm longitudinal dimension of the acceleration bucket ensures negligible phase slippage of electron bunches with a typical 10^−3^ energy spread, while the 100 μm transverse dimensions of the accelerating field allow for the entire injected electron bunch to be captured and accelerated without transverse phase-space degradation.

### MV m^−1^ field strengths for particle acceleration

**Fig. 5:**
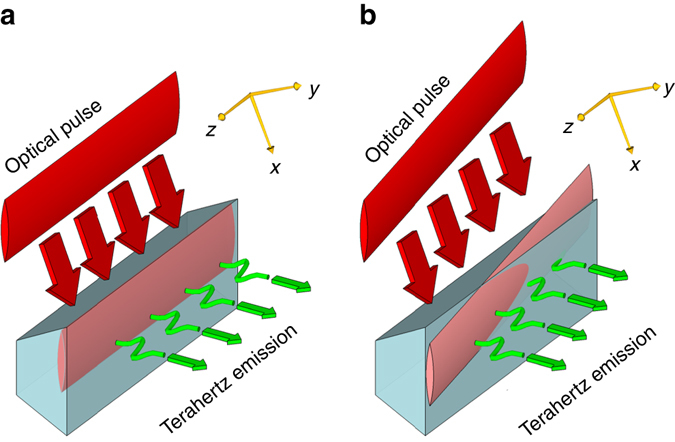
Laser pulse-front tilt for terahertz generation in LiNbO_3_. **a** Traditional configuration, with a pulse-front tilt in the *x*,*y* direction matched to the Cherenkov angle. **b** Modified configuration which maintains the Cherenkov tilt angle in the *x*,*y* plane, with an additional tilt component in the *x–z* plane, resulting in an effective travelling source in the *z*-direction

The above measurements with interdigitated PCA and ZnTe optical rectification sources serve to illustrate the concept of a single-cycle source with longitudinal polarisation and sub-luminal dispersionless propagation. Neither of these optical to terahertz mechanisms or media are however capable of generating the multi-MV m^−1^ field strengths sought for high-gradient relativistic particle acceleration. To achieve high field strengths the same travelling source concept has been adapted to work with a LiNbO_3_ nonlinear medium, a crystal that is capable of greater than 100 MV m^−1^ field strengths^50^. A significant hurdle is encountered in using this established material for high-field terahertz generation, in that non-colinear propagation of terahertz and optical frequency waves is required to satisfy the terahertz and optical phase-matching^51^. For LiNbO_3_ the optical group velocity exceeds the phase velocity of the generated terahertz radiation by a factor of approximately 2.5. As a consequence the terahertz radiation is generated in an obliquely propagating Cherenkov cone centred around the laser propagation axis. To allow efficient generation from a transversely extended optical beam a temporal-spatial correlation is introduced in the optical excitation pulse, with the correlation such that the locally produced Cherenkov cones add constructively to produce plane-wave emission in a specific Cherenkov direction^51, 52^. To provide a $v_\phi ^{{\rm{eff}}} < c$ travelling source in LiNbO_3_, we have developed a scheme that maintains the standard Cherenkov pulse-front tilt in one plane, while adding spatially dependent time delays in the orthogonal direction. As shown in Fig. 5, the LiNbO_3_ crystal is oriented with the standard Cherenkov tilt in the *x*,*y* plane, with the additional time delays in the *x–z* plane giving rise to a propagating source in the *z*-direction. While the *z*-direction delays could in principle be introduced by an optical staircase in the laser beam, here we achieve the continuous rather than discretised propagation through introduction of an additional component of the pulse-front tilt in the *x–z* plane. An *x,y* tilt of 24° corresponds to a propagation velocity of $v_\phi ^{\rm{z}} = c$. We refer to this arrangement as a ‘double-tilt’ configuration, although clearly each of the tilts are actually orthogonal components of a single (larger) pulse-front tilt projected into the exit plane of the LiNbO_3_ crystal.

**Fig. 6:**
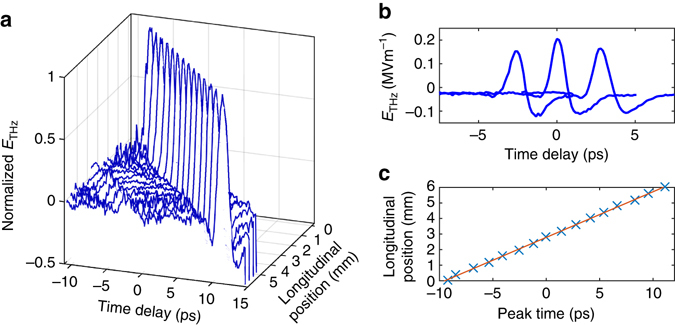
Measured terahertz pulse propagation in a plane parallel to the LiNbO_3_ emitting surface. **a** Electric field temporal profiles as a function of position along the LiNbO_3_ surface. The field strength is normalised to the peak strength at each measurement position. **b** Example terahertz electric field profiles taken from the same data set shown in **a**, without normalisation. **c** Arrival time of the peak electric field as a function of longitudinal position. The *red line* is linear fit to the peak positions and corresponds to a phase and group velocity of 0.98*c*

As constructed, the double-tilt arrangement seeks to maintain the terahertz generation efficiency enhancement that comes from transverse velocity matching to the Cherenkov angle. While such an enhancement will be expected to be maintained for a propagation delays introduced by a sufficiently coarse staircase delay optical element, the effect of a continuous delay from the orthogonal tilt component is less clear. We have carried out three dimensional FDTD simulations of the terahertz generation with single-tilt and double-tilt optical propagation and have confirmed that for our LiNbO_3_ parameters the source-propagation tilt has a negligible effect on the efficiency of the terahertz field strength in the emission surface.

The double-tilt approach has been demonstrated through an optical arrangement similar to that employed for the ZnTe and PCA measurements. The combined pulse-front tilt was maintained in the horizontal lab-frame horizontal plane, with the consequence that the transverse and longitudinal (effective propagation) directions, and the emission surface of the LiNbO_3_ were oriented out of the laboratory frame horizontal or vertical planes. As for the ZnTe and PCA measurements, a temporal and spatial mapping of the fields near the surface was obtained with EO sampling. The transverse intensity profile of the optical beam was also shaped with cylindrical telescopes so that following oblique incidence on the grating and projection onto the out-of-plane oriented crystal surface, the terahertz source dimensions were ~15 mm in the propagating source longitudinal direction, and 2 mm in the orthogonal transverse (Cherenkov matched) direction.

A temporal and spatial map of the terahertz field 500 μm from the LiNbO_3_ surface was measured with electro-optic sampling and is summarised in the data of Fig. 6c–e. In this data set the single-cycle terahertz pulse is travelling at 0.98*c*, as determined through the time-delay of the peak longitudinal electric field as a function of longitudinal position across the emitting surface. The laser pulse energy incident on the LiNbO_3_ terahertz generating crystal was limited by the available laser system, and diffraction grating and optical losses, to 600 μJ. The transverse optical pulse shaping increased the optical pump energy density in the nonlinear LiNbO_3_ while maintaining the desired propagation distance of approximately 10 mm. Absolute electric field strength of a terahertz pulse can in principle be determined in EO sampling, through calibration of the EO signal into absolute polarisation rotation of the optical probe together with knowledge of the phase-matching and *χ* ^(2)^ nonlinear response of the EO detection crystal^36^. Where the terahertz and optical probe do not propagate collinearly in the detection crystal, the experimental response function is further subject to angular separation of the optical probe and nonlinear generated optical waves^53^. The physical separation of the optical waves in propagation from detection crystal to the photodiode detectors will give rise to an underestimate of the terahertz electric field strengths. The field reduction arising from this physical separation of optical waves is extremely challenging to objectively determine, and here we have instead provided a lower bound measurement of the electric field, based on an assumption of collinear terahertz and optical probe waves. With our modest laser powers, we obtain a lower bound measured field strength of 0.20 MV m^−1^. Based on the established capability of LiNbO_3_ we believe the true field strength could be significantly higher, and that with improvements in laser system field strengths approaching 10 MV m^−1^ may be readily obtained. Such accelerating field gradients would already be comparable to state-of-the-art superconducting accelerators such as the European X-ray free-electron laser. Even higher field strengths may be achievable through use of alternative nonlinear materials with demonstrated high-efficiency and high-energy generation capability^26–28^.

## Discussion

We have described and demonstrated a method for generating single-cycle terahertz pulses that propagate with an effective sub-luminal velocity, and without distortion of the single-cycle field profile during propagation. The electric field polarisation is constructed with a component in the longitudinal or propagation direction as required for acceleration of charged-particle beams. The transverse electric and magnetic fields that arise from the spatial and temporal gradients of the travelling source provide a focusing structure analogous to RF particle accelerators, but with a 1000-fold reduction in spatial scale. The 1 ps duration of the terahertz pulse is six orders of magnitude smaller than the μs cavity fill-time of conventional RF accelerating structures. The 10^6^ reduction in the time-integrated energy content of the electromagnetic field while maintaining field overlap with particle beams offers the potential for dramatic reductions in the power requirement of high energy particle accelerators. While the particular demonstrations of single-cycle sub-luminal propagation presented here do not have the accelerating field strengths desired for immediate applications, sufficiently high field strengths are within reach of current sources. To be applied to acceleration of beams for ultrafast electron diffraction, energy gain of between 100 keV and 5 MeV is required, for bunch charges of less than 1pC, corresponding to $\ll 5$ μJ of energy transfer to the electron beam; as context, it is noted that 900 μJ, and GV m^−1^ terahertz sources have already been demonstrated^26–28^. Higher energy beams are required for the terahertz travelling source acceleration concept to be applied as a driver for free-electron lasers. A soft X-ray free-electron laser may be expected to operate with 1 GeV, 10 pC bunches, corresponding to a total beam energy of 10 mJ. While terahertz sources sufficiently energetic to provide this level of beam energy are not currently available, a principle benefit of our travelling source concept is that it is directly scalable with length, with no dispersion derived limitations. With rapid developments occurring in the efficiency and energy of nonlinear sources, a sub-luminal travelling terahertz source offers a promising route to an all-laser driven free-electron laser.

## Methods

### Pump source

The Ti:Sapphire regenerative amplifier laser system used to generate terahertz radiation produced pulses with an energy of up to 2 mJ and a pulse duration of ~45 fs at a repetition rate of 1 kHz, with a central wavelength of 800 nm. Three different generation media were used; a 0.5 mm thick (110)-cut ZnTe crystal, a commercially-available interdigitated photoconductive antenna (TeraSED10, Laser Quantum Ltd.), and a 6% magnesium-oxide doped stoichiometric lithium niobate crystal. Further details of the interdigitated photoconductive antenna (PCA) can be found in ref. ^54^.

### Terahertz frequency generation with PCA and ZnTe

The interdigitated photoconductive antenna was biased with 25 V pulses at a repetition rate of 500 Hz with a duty cycle of ~10% and photo-excited with 25 μJ pulses at a repetition rate of 1 kHz from the regenerative amplifier laser system. The ZnTe crystal was pumped with 500 μJ pulses and an optical chopper was used to reduce the repetition rate to 500 Hz for lock-in detection. A filter was used to block the 800 nm pump light from being transmitted to the ZnTe detection crystal. Diffraction gratings with 1200 lines/mm and 600 lines/mm were used in a 4 f imaging arrangement (consisting of two 250 mm focal length cylindrical lenses) to produce a range of pulse-front-tilt angles within the generation medium. A 400 mm cylindrical lens was used to achieve an increase in the excitation energy density at the terahertz generation point without affecting the pulse-front-tilt angle.

### Terahertz frequency generation with LiNbO_3_

**Fig. 7:**
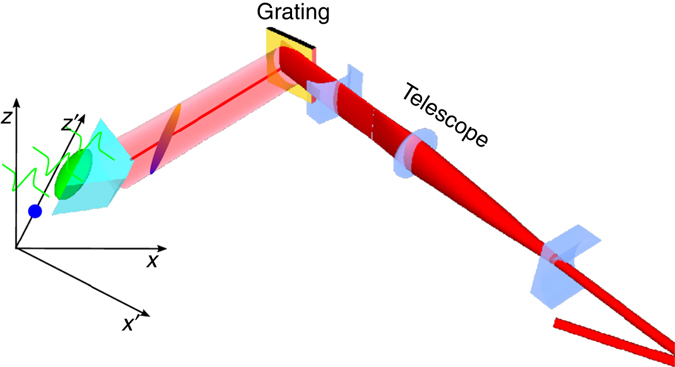
Schematic arrangement for matching the transverse laser profile to a travelling source geometry in LiNbO_3_. The cylindrical lens telescope shapes the laser transverse profile to an elongated elliptical beam on the LiNbO_3_ emission surface. The LiNbO_3_ crystal is rotated with respect to the diffraction plane of the grating to provide components of the pulse-front tilt in both the Cherenkov phase-matching and travelling source directions. For clarity optical elements between the diffraction grating and crystal are not shown

The lithium niobate crystal was pumped at 500 Hz with 600 μJ pulses after losses from optics and the diffraction grating. A 1200 lines/mm diffraction grating was used to produce a pulse-front tilt of 43.8° and was imaged to the crystal using a single lens giving a magnification factor of 0.21 to give a total pulse-front tilt of 77.6° (63.8° inside the LiNbO_3_). A zero-order half-wave plate was used to rotate the polarisation of the first-order diffracted beam in order to match the *z*-axis of the lithium niobate crystal. The crystal was rotated by an angle of ~12.7° to give a phase-matching component of the tilt of 77.3° (63.0° inside the crystal) and a velocity generating component of 45.0° (24.2° inside), giving an expected velocity of approximately 1.0*c*. As shown schematically in Fig. 7, The pump beam was shaped into an ellipse with the major axis aligned along the direction of travel of the generated terahertz wave. This was achieved using a telescope consisting of a two −50 mm focal length cylindrical lenses and a 250 mm spherical lens. These were used to produce a magnification factor of 5 in the vertical axis and 1/5 in the horizontal axis. The two cylindrical lenses were rotated about the pump propagation axis by ~3.6° in order to match the ellipse rotation to that of the lithium niobate crystal. The 3.6° rotation, along with projections onto the diffraction grating, produced a final elliptical beam with a rotation of ~12.7°.

### EO detection

In the PCA and ZnTe experiments, a 1 mm thick (110)-cut ZnTe crystal was used to detect the generated terahertz radiation with a separation of ~500 μm between the generation medium and the detection crystal. In the LiNbO_3_ experiments, a 0.5 mm thick (110)-cut GaP crystal was used with a separation of ~300 μm. In both cases, a balanced electro-optical detection scheme was used to measure the generated terahertz electric fields. The reflection of the probe pulse off of the inner surface of the detection crystal acquired a polarisation change which was proportional to the terahertz electric field strength. The probe pulse was then separated into two orthogonal polarisation states using a polarising beam splitter and the intensities of each were measured using balanced photodiode detection scheme. A lock-in amplifier was used to measure the voltage change from the two photodiodes at the 500 Hz frequency of the pump pulse or the PCA bias voltage. The probe beam was focused to a ≲100 μm diameter spot at the detection crystal and its position could be translated horizontally and vertically, while maintaining a constant path length, using translation stages in order to measure the terahertz electric fields at different spatial positions over the crystal surface. A calibration of the probe beam translation stages was performed by attenuating the probe pulse and taking images of the probe beam displacements on the surface of the detection crystal using a CCD camera.

### FDTD simulations and particle evolution

The finite difference time domain (FDTD) method is used to determine the fields as a function of time. The travelling bipolar terahertz source is embedded within the dielectric material ($\epsilon$ = 9${\epsilon _0}$) with velocity *β*_s_ = 1 − 5 × 10^−3^, matching a *γ* = 10 (*U* ≈ 5 MeV) electron beam velocity. In line with the optical rectification generation process there is no magnetic field source and the electric field of the source term is polarised purely in the *z*-direction, which also corresponds to our chosen source propagation direction. The spatial grid for the simulations of Figs. 2 and 4 is 5 μm and the time step is 12 fs.

The particle phase-space evolution was obtained through numerical solution of the relativistic equation of motion

1 $$\frac{{{\rm{d}}{\boldsymbol{\beta }}}}{{{\rm{d}}t}} = - \frac{e}{{{m_{\rm{e}}}c}}\sqrt {1 - {{\left| \beta \right|}^2}} \left[ {{\bf{E}} + c{\boldsymbol{\beta }} \times {\bf{B}} - {\boldsymbol{\beta }}\left[ {{\boldsymbol{\beta }} \cdot {\bf{E}}} \right]} \right].$$

A fourth-order Runge–Kutta algorithm was embedded within the FDTD field evaluation algorithm, with particle velocities and positions updated at each time step of the FDTD code. The position and momentum of 1000 electrons were tracked as they progated through the double-sided field structure. The initial injected electron beam parameters are chosen to be representative of the VELA linear electron accelerator^55^ with electron momentum *p* = 5.2 MeV/c (*β* = 0.996).

### Data availability

The data associated with the paper are openly available from the Mendeley Data Repository at: http://dx.doi.org/10.17632/5gd9vymhfb.1.
