# Author Correction: Demonstration of sub-luminal propagation of single-cycle terahertz pulses for particle acceleration

**DOI:** 10.1038/s41467-017-01653-7

**Published:** 2017-11-23

**Authors:** D. A. Walsh, D. S. Lake, E. W. Snedden, M. J. Cliffe, D. M. Graham, S. P. Jamison

**Affiliations:** 1 Accelerator Science and Technology Centre, Science and Technology Facilities Council, Daresbury Laboratory, Keckwick Lane, Daresbury, Warrington WA4 4AD UK; 2 The Cockcroft Institute, Sci-Tech Daresbury, Keckwick Lane, Daresbury, Warrington WA4 4AD UK; 3 School of Physics and Astronomy & Photon Science Institute, The University of Manchester, Manchester, M13 9PL UK

*Nature Communications* **8**:421 doi:10.1038/s41467-017-00490-y; Article published online 4 September 2017

The original version of this Article contained an error in the abstract, referring to “multi-megawatt-per-metre” instead of “multi-megavolt-per-metre”. This has now been corrected in both the PDF and HTML versions of the Article.

